# Circadian rest‐activity rhythm as an objective biomarker of patient‐reported outcomes in patients with advanced cancer

**DOI:** 10.1002/cam4.1711

**Published:** 2018-08-07

**Authors:** Pasquale F. Innominato, Sandra Komarzynski, Oxana G. Palesh, Robert Dallmann, Georg A. Bjarnason, Sylvie Giacchetti, Ayhan Ulusakarya, Mohamed Bouchahda, Mazen Haydar, Annabelle Ballesta, Abdoulaye Karaboué, Nicholas I. Wreglesworth, David Spiegel, Francis A. Lévi

**Affiliations:** ^1^ North Wales Cancer Centre Ysbyty Gwynedd Betsi Cadwaladr University Health Board Bangor UK; ^2^ Cancer Chronotherapy Team Cancer Research Centre Warwick Medical School Coventry UK; ^3^ Unit 935 French National Institute for Health and Medical Research (INSERM) Villejuif France; ^4^ Department of Psychiatry and Behavioral Sciences Stanford University Stanford California; ^5^ Stanford Cancer Institute Stanford School of Medicine Stanford California; ^6^ Sunnybrook Odette Cancer Centre University of Toronto Toronto ON Canada; ^7^ Department of Oncology Saint Louis Hospital Public Hospitals of Paris (AP‐HP) Paris France; ^8^ Chronotherapy Unit Department of Medical Oncology Paul Brousse Hospital Public Hospitals of Paris (AP‐HP) Villejuif France; ^9^ Mousseau Clinics Evry France; ^10^ Warwick Mathematics Institute University of Warwick Coventry UK; ^11^ AK‐SCIENCE, Research and Therapeutic Innovation Vitry‐sur‐Seine France

**Keywords:** actigraphy, Circadian, patient‐reported outcome, quality of life, symptom

## Abstract

**Background:**

Psychosocial symptoms often cluster together, are refractory to treatment, and impair health‐related quality of life (HR‐QoL) in cancer patients. The contribution of circadian rhythm alterations to systemic symptoms has been overlooked in cancer, despite a causal link shown under jet lag and shift work conditions. We investigated whether the circadian rest‐activity rhythm provides a reliable and objective estimate of the most frequent patient‐reported outcome measures (PROMs).

**Methods:**

Two datasets were used, each involving concomitant 3‐day time series of wrist actigraphy and HR‐QoL questionnaires: EORTC QLQ‐C30 was completed once by 237 patients with metastatic colorectal cancer; MD Anderson Symptom Inventory (MDASI) was completed daily by 31 patients with advanced cancer on continuous actigraphy monitoring, providing 1015 paired data points. Circadian function was assessed using the clinically validated dichotomy index *I* < *O*. Nonparametric tests compared PROMs and *I* < *O*. Effect sizes were computed. Sensitivity subgroup and temporal dynamics analyses were also performed.

**Results:**

*I* < *O* values were significantly lower with increasing symptom severity and worsening HR‐QoL domains. Fatigue and anorexia were worse in patients with circadian disruption. The differences were both statistically and clinically significant (*P* < 0.001; *d* ≥ 0.33). Physical and social functioning, and global quality/enjoyment of life were significantly better in patients with robust circadian rhythm (*P* < 0.001; *d* ≥ 0.26). Sensitivity analyses validated these findings.

**Conclusion:**

Objectively determined circadian disruption was consistently and robustly associated with clinically meaningfully severe fatigue, anorexia, and interference with physical and social functioning. This supports an important role of the circadian system in the determination of cancer patients’ HR‐QoL and symptoms that deserves therapeutic exploitation.

## 1 INTRODUCTION

Currently, one in eight adults carries wearable “well‐being” activity monitors,^1^ with some 50 million such units been sold in the United States in 2016.^2^ This positive perception of e‐Health devices in the general population represents an opportunity for implementing objective measures of physiology and behavior complementing the assessments of symptoms and health‐related quality of life (HR‐QoL), especially in cancer patients.^3^ Indeed, systemic psychosocial symptoms are usually subjectively rated using validated questionnaires evaluating patient‐reported outcome measures (PROMs).^4^ More specifically, fatigue, sleep disturbance, depression, and anorexia represent the most frequent psychosocial complaints of cancer patients.^5^ Extensive research has shown that cancer patients tend to display multiple systemic symptoms that often cluster together.^6^ Fatigue, anorexia, and affective disorders can also arise as a consequence of anticancer treatment, suggesting shared physiopathological mechanisms.^7^ Such systemic ailments also reveal the disruption of those body clocks that time behavioral and cellular activities along the 24‐hour cycle, for example, as a consequence of jet lag or shift work.^8^, ^9^, ^10^, ^11^ All living beings, from unicellular organisms to humans, are endowed with endogenous biological clocks that enable living organisms to anticipate cyclic environmental changes and coordinate physiological events.^12^ The mammalian circadian timing system (CTS) is hierarchically organized and temporally controlled, and it coordinates several physiological processes, at whole‐body, cellular, down to molecular, levels.^13^, ^14^ In particular, sleep‐wake cycles, physical and mental performance, as well as appetite, are modulated along the 24 hours by the CTS.^15^, ^16^ As altered patterns have been described for several circadian rhythms in cancer patients,^15^, ^17^, ^18^ we hypothesized that systemic symptoms would be more severe in patients with circadian disruption. Two independent datasets were used to test this hypothesis. Based on the convenience of its noninvasive methodology and previous reports in smaller cohorts showing an association with fatigue and sleep problems,^19^, ^20^, ^21^, ^22^, ^23^, ^24^, ^25^ we selected the circadian rest‐activity rhythm as measured by wrist actigraphy.^26^, ^27^ Wrist actigraphy has been validated as an objective biomarker of circadian function.^28^ Finally, objective actigraphy data were correlated with selected subjective PROMs, including systemic symptoms and HR‐QoL domains.

## 2 PATIENTS AND METHODS

### 2.1 Study populations

For this study, we used datasets from two patient cohorts, involving different PROMs tools and methodologies. The original data were available for both objectively assessed circadian rest‐activity rhythm (wrist actigraphy)^28^ and subjectively rated symptoms as well as HR‐QoL (with validated questionnaires) from the same patients. For both patient cohorts, approval had been obtained from the appropriate ethical review boards, and patients had provided signed informed consent.^29^, ^30^, ^31^

#### 2.1.1 Cohort #1

The first set was composed of patients with metastatic colorectal cancer, who were not at time of monitoring having anticancer treatment and had a WHO performance status of 0, 1, or 2. They had participated to either a monocentric study from May 1994 to January 1997 (Chronotherapy Unit, Department of Medical Oncology, Paul Brousse University Hospital, Villejuif, France)^31^ or to a companion study of an international randomized trial involving nine institutions in four countries, from August 1999 to February 2002.^29^

Patients in both studies underwent wrist actigraphy monitoring for 72 consecutive hours (Mini‐motionlogger, Ambulatory Monitoring Inc., Ardsley, NY, USA) and completed the European Organization for Research and treatment of Cancer Quality of Life Core (EORTC QLQ‐C30 v2.0) questionnaire.^32^ This internationally validated 30‐item questionnaire incorporates eight symptoms, five functioning domains and one global QoL scale. All scores were transformed to a 0‐100 scale, according to the recommended EORTC procedures. For the symptom scales, low scores corresponded to mild symptoms, whereas for the global QoL and its domains scales, low scores indicated poor functioning.^32^ In the analysis, we selected the systemic symptoms (fatigue, anorexia, sleep problems, pain), global QoL, and the functioning domains (physical, role, and social), corresponding to the items in the M.D. Anderson Symptom Inventory (MDASI).^33^

#### 2.1.2 Cohort #2

The second set included patients with histologically proven advanced or metastatic cancer requiring medical treatment. The patients had participated to the pilot project on multidimensional tele‐monitoring from home performed at the Chronotherapy Unit, Department of Medical Oncology, Paul Brousse University Hospital, Villejuif, France, between April 2012 and July 2013, within the framework of the inCASA European project (FP7).^30^ The patients were equipped with a home‐based platform for multidimensional tele‐monitoring over at least 30 consecutive days. This remote surveillance included continuous wrist actigraphy (Micro‐motionlogger, Ambulatory Monitoring Inc., Ardsley, NY, USA) and once‐daily completion of an electronic version of the MDASI questionnaire^33^ using an interactive screen. This 19‐item validated questionnaire evaluates the severity (from 0 to 10) of 13 core symptoms and of their interference with six activities of daily living. Large scores for each item indicate severe symptoms.^33^ Patients were monitored while being treated with chemotherapy, as indicated according to their medical condition.^30^ Hence, this data‐dense study provided dynamic patterns of circadian rest‐activity rhythm and PROMs. As both circadian rest‐activity rhythm and symptom severity on chemotherapy are not stationary and present temporary variations,^34^ we did not pool all data from a single patient over the monitoring span (exceeding 30 days). Instead, we analyzed actigraphy data over 72 consecutive hours, with 3‐day sliding windows and a 1‐day shift, throughout the time series in each patient, and used individual daily data in MDASI items scores, as previously described.^30^ Hence, this dataset provided a larger amount of data than the actual number of patients. For the analysis, we selected the systemic symptoms (fatigue, anorexia, sleep disturbance, pain) and the interference items (general activity, work, relations with others, and enjoyment of life), corresponding to the items from the EORTC questionnaire.

Among both cohorts, there were no uncontrolled metabolic, endocrine, or autoimmune diseases and no symptomatic brain metastases (the details are provided in the original papers^29^, ^30^, ^31^).

Figure S1 displays the study flowchart.

### 2.2 Wrist actigraphy

The wrist‐worn accelerometers used in both cohorts were manufactured by the same company (Ambulatory Monitoring Inc., Ardsley, NY, USA), which provided also the dedicated analytical software (Action 4). In the second cohort study, the patients downloaded and tele‐transmitted the rest‐activity data collected over the past 24 hours using the home Internet platform.^30^ For both cohorts, the epoch length for data collection was set at 1 minute, according to common practice.^28^ The actigraph collects and stores the number of wrist accelerations per minute, across the three axes, from the nondominant arm. The pattern of accelerations over time is then analyzed to compute pertinent parameters for assessing circadian rest‐activity rhythm, over 72 consecutive hours, as recommended.^28^

### 2.3 Statistical analyses

We selected the dichotomy index *I* < *O* as the most clinically relevant actigraphy parameter, based on prior studies from others and ourselves.^29^, ^30^, ^31^, ^35^, ^36^, ^37^, ^38^ *I* < *O* is the percentage of activity counts per minute when the patient is in bed at night with values lower than the median activity count when the patient is out of bed during the day.^39^ Hence, it can range from 0% to 100%. In case of restful sleep at night and lively activity during the day, a robust and prominent circadian rhythm is present, and *I* < *O* will be close to 100%.^39^ To categorize patients with circadian disruption or not, we used the cut‐off point for *I* < *O* of 97.5%, as previously identified and validated.^36^, ^37^ Thus, when *I* < *O* was lower or equal to 97.5% we estimated that circadian rest‐activity rhythm disruption was present, whereas this rhythm was deemed maintained when *I* < *O* was greater than 97.5%.

Summary statistics were computed to describe the distribution of *I* < *O* values (median and interquartile range) and of PROMs (means, SD, and SEM). First, we categorized the PROMs items into terciles, and compared the distributions of *I* < *O* among the terciles with the Jonkheere‐Terpstra test. Secondly, we defined two categories of patients using the previously established cut‐off point of 97.5% for *I* < *O* as a marker of circadian disruption. The distribution of the EORTC or the MDASI items were compared between the two groups, using an independent sample *t* test. We also evaluated the effect size of the difference in PROMs scores between the two groups by computing Cohen's *d*, with a threshold for clinically meaningful difference set at *d* ≥ 0.25. Additionally, we assessed the clinical relevance of the absolute differences based on previously identified thresholds: 10 points for the EORTC questionnaire and one point for the MDASI scale, respectively.^40^, ^41^ We used also the nonparametric Mann‐Whitney *U* test to compare questionnaires items according to *I* < *O* category, as sensitivity analysis. For cohort #1, we performed subgroup analysis according to sex, PS, and age, using the same methodology. For cohort #2, we performed additional comparisons of the dynamic patterns of PROMs and *I* < *O*. Thus, we computed the differences in *I* < *O* between each day and the previous one, with a sliding window approach. The distribution of changes in selected PROMs was compared in each of the three subgroups defined by the terciles of the changes in *I* < *O* (improved, stable, worsened) with Wilcoxon signed‐rank test.

Moreover, Spearman's rank correlations between *I* < *O* and selected PROMs indices were computed for each cohort. Finally, we built a multivariate linear regression model with global quality of life (for cohort #1) or interference with enjoyment of life (for cohort #2) as dependent variables, and all the other selected PROMs of each questionnaire and *I* < *O* as independent variables, to assess the objective, additional information about HR‐QoL provided by *I* < *O*. Analyses were performed using PASW v24 (SPSS, IBM Inc., Chicago, IL, USA) and Stata v14 (StataCorp LLC, College Station, TX, USA) software packages. The threshold for statistical significance was set at *P* ≤ 0.005, correcting for multiple comparisons.

## 3 RESULTS

### 3.1 Study populations

Study cohort #1 included 237 patients with metastatic colorectal cancer, who completed the EORTC QLQ‐C30 questionnaire and underwent 3‐day wrist actigraphy recording (Table S1). Study cohort #2 included 31 patients, mostly suffering from advanced or metastatic gastro‐intestinal malignancy and having 1015 valid dyads of 3‐day wrist actigraphy recordings and daily completion of the MDASI questionnaire, at the intermediate day of the 3‐day actigraphy sliding window (Table S1). Altogether, nearly 90% of the patients in either population had a performance status of 0 or 1, despite advanced disease (Table S1).^29^, ^30^, ^31^

### 3.2 Descriptive statistics

Table S2 provides mean and SD values for EORTC symptom scales and quality of life domains (range, 0 to 100) in cohort #1, and for MDASI items (range, 0 to 10) in cohort #2 (Table S2).

The cut‐off points for the terciles of PROMs are detailed in Table S3.

In both populations, the distribution of *I* < *O* values was skewed toward high values, as in previous reports^38^, ^42^ (Figure S2). Median values, in both cases, were close to 97.5%, formerly reported as a clinically meaningful cut‐off point^36^, ^37^: 96.9% [1st and 3rd quartiles: 93.6%‐99.1%] for cohort #1, and 98.0% [95.8‐99.0] for cohort #2 (Figure S1). Thus, the proportion of instances with circadian disruption (ie, with *I* < *O* ≤ 97.5%) was 54.9% in cohort #1 and 44.4% in cohort #2.

### 3.3 Comparative analyses of wrist actigraphy monitoring and questionnaires

**Figure 1:**
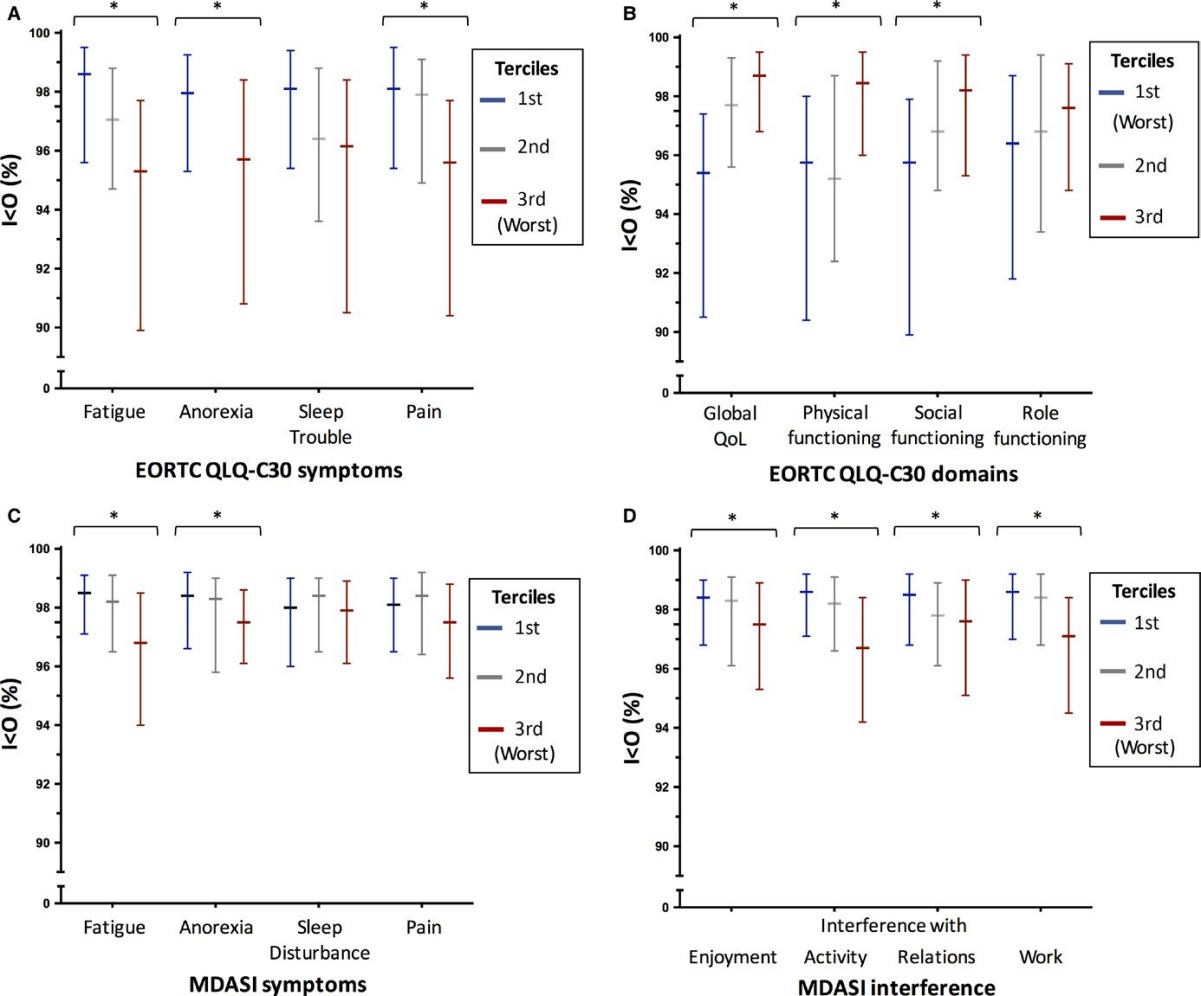
Median (and interquartile range) *I* < *O* values in the subgroups defined by the terciles of the PROMs items from the EORTC (panels A and B) and MDASI (panels C and D) questionnaires: blue, first; gray, second; red, third. In all cases, the higher the tercile, the more severe the symptom, except for the EORTC QLQ‐C30 domains (panel B), in which the higher the tercile, the better the quality of life and the functioning. In all cases, stars indicate *P *<* *0.0001. Other nonsignificant *P* values are detailed in the Section 3

In cohort #1, *I* < *O* significantly decreased with increasing severity of fatigue (*P* < 0.0001), anorexia (*P* < 0.0001), pain (*P* < 0.0001), and sleep trouble (*P* = 0.003) (Figure 1A). In contrast, *I* < *O* significantly increased with greater values of global quality of life (*P* < 0.0001), physical (*P* < 0.0001), and social (*P* < 0.0001) functioning, but not role (*P* = 0.02) functioning (Figure 1B). In cohort #2, significantly lower *I* < *O* values were observed with gradually more severe fatigue and anorexia, as well as interference with enjoyment of life, activity, relations with others, and work (all *P* < 0.0001), whereas differences were not significant for sleep disturbance (*P* = 0.56) and pain (*P* = 0.009; Figure 1C,D).

The comparison of PROMs as a function of circadian disruption (*I* < *O* ≤ 97.5%) or robustness (*I* < *O* > 97.5%) yielded similar results. Thus, in cohort #1, patients with circadian disruption complained of statistically more severe fatigue (*P* < 0.0001), anorexia (*P* < 0.0001), and pain (*P* < 0.0001), yet only a nonsignificant trend (*P* > 0.005) was found for sleep trouble (*P* = 0.009). In cohort #2, fatigue (*P* < 0.0001) and anorexia (*P* < 0.0001) were also rated as significantly more severe when *I* < *O* ≤ 97.5%, whereas sleep disturbance (*P* = 0.61) and pain (*P* = 0.02) were not.

**Figure 2:**
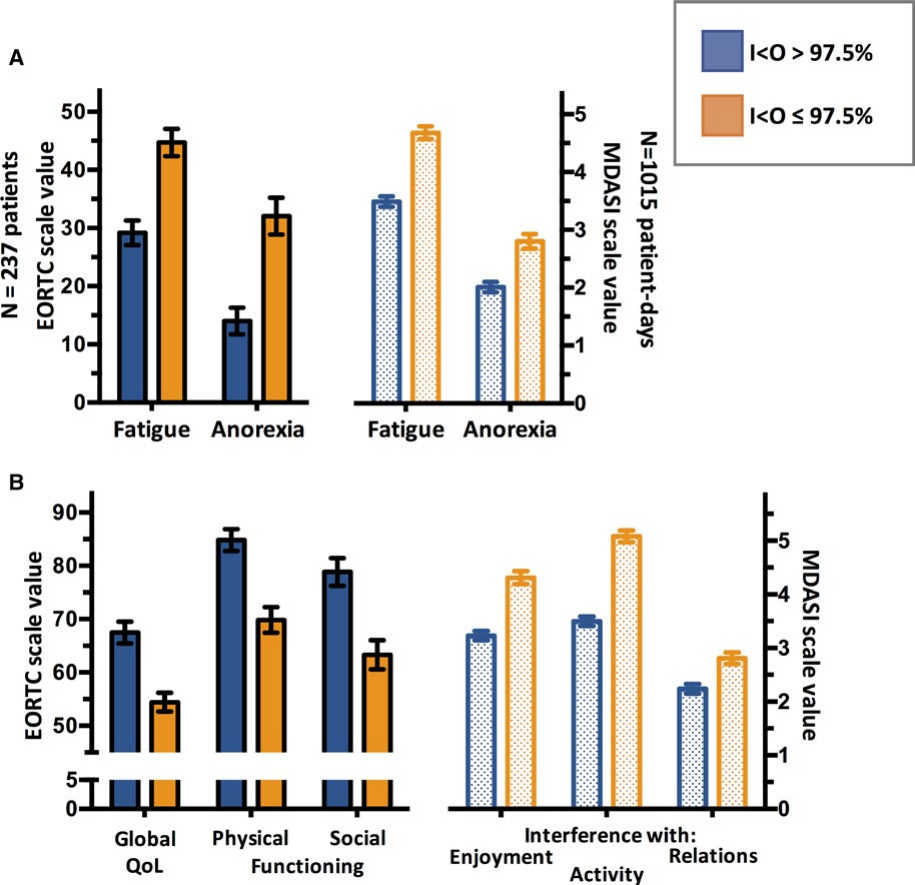
Mean (±SEM) values PROMs indices according to high (blue) or low (orange) *I* < *O*, indicating robust and disrupted circadian rest‐activity rhythm, respectively. For symptoms (panel A), in both scales, higher values imply worse symptom severity (range for EORTC: 0‐100; for MDASI: 0‐10). In panel B, for the EORTC scale, higher values designate better quality of life domains (range: 0‐100), while for MDASI, lower values imply less intense interference (range: 0‐10). For all comparisons, *P* < 0.001

In cohort #1, global quality of life (*P* < 0.0001), physical functioning (*P* < 0.0001), and social functioning (*P* < 0.0001) were rated as significantly poorer by patients with *I* < *O* ≤ 97.5% as compared to those with higher *I* < *O* values, whereas role functioning (*P* = 0.04) was not. In cohort #2, instances with circadian disruption were significantly associated with greater interference with enjoyment of life, activity, relations with others, and work (all *P* < 0.0001). The associated effect sizes were of intermediate magnitude in both populations for the statistically different items (Table 1). Figure 2 displays the mean (±SEM) values for both populations for selected corresponding symptoms (panel A) or functioning/interference items (panel B).

**Table 1:** Differences in means and associated effect sizes (Cohen's *d* values) between subgroups with circadian disruption (*I* < *O* ≤ 97.5%) and those with robust circadian function (*I* < *O* > 97.5%) for all tested subjective items in each study population

	EORTC items [0‐100] (cohort #1)				MDASI items [0‐10] (cohort #2)		
	Difference	Cohen's *d*	*P*		Difference	Cohen's *d*	*P*
Fatigue	15.5	0.63	<0.001	Fatigue	1.19	0.54	<0.001
Anorexia	18.0	0.58	<0.001	Anorexia	0.79	0.33	<0.001
Sleep trouble	10.3	0.34	0.009	Sleep disturbance	0.14	0.07	0.61
Pain	14.0	0.56	<0.001	Pain	0.53	0.20	0.02
Global Quality of Life	−13.0	0.64	<0.001	Interference with Enjoyment of Life	1.08	0.48	<0.001
Physical Functioning	−15.0	0.61	<0.001	Interference with Activity	1.58	0.73	<0.001
Social Functioning	−15.5	0.54	<0.001	Interference with Relations with Others	0.57	0.26	<0.001
Role Functioning	−10.1	0.31	0.04	Interference with Work	1.57	0.68	<0.001

Positive differences represent higher values when *I* < *O* ≤ 97.5%.

Correlative analyses further confirmed a negative association between *I* < *O* and the severity of self‐rated fatigue and anorexia, in both populations (Table 2). *I* < *O* was also found negatively correlated with pain and sleep trouble, and positively correlated with global quality of life, physical, social, and role functioning in cohort #1. In cohort #2, *I* < *O* was negatively correlated also with pain, and with interference with enjoyment of life, activity, relations with others and work, while it was positively correlated with interference with mood. The absolute values of the correlation coefficients were mostly ≥0.2, yet invariably <0.4 (Table 2).

**Table 2:** Spearman's rank correlations between *I* < *O* and selected items from EORTC QLQ‐C30 and MDASI questionnaires

Cohort #1	*r*	*P*	Cohort #2	*r*	*P*
EORTC items			MDASI items		
Fatigue	−0.33	<0.001	Fatigue	−0.26	<0.001
Anorexia	−0.29	<0.001	Anorexia	−0.17	<0.001
Sleep trouble	−0.20	0.002	Sleep disturbance	−0.03	0.28
Pain	−0.31	<0.001	Pain	−0.10	0.001
Global quality of life	0.33	<0.001	Interference with enjoyment of life	−0.19	<0.001
Physical functioning	0.36	<0.001	Interference with activity	−0.32	<0.001
Social functioning	0.28	<0.001	Interference with relations with others	−0.12	<0.001
Role functioning	0.19	0.004	Interference with work	−0.32	<0.001

**Figure 3:**
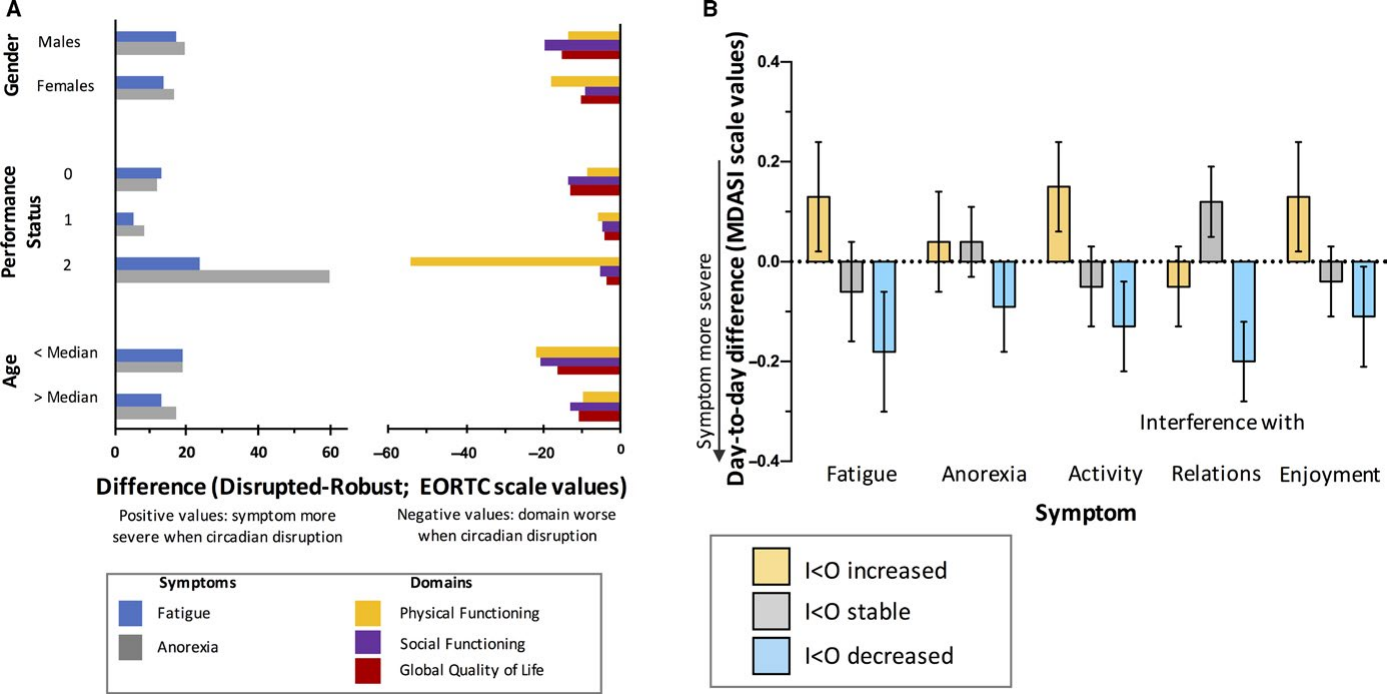
Sensitivity subgroup and dynamic analyses. Panel A (cohort #1): difference in mean EORTC item values between patients with circadian disruption (*I* < *O* ≤ 97.5%) and circadian robustness, in the subgroups defined by sex, PS, and age. For the symptoms, positive values reflect worse severity in patients with circadian disruption. For the domains, negative values indicate poorer quality of life in patients with circadian disruption. Panel B (cohort #2): mean (±SEM) day‐to‐day changes in MDASI scores in the subgroups of cases defined by improved (yellow), stable (gray), or worsened (blue) circadian function (increased, unchanged, or decreased *I* < *O*, respectively). Changes to more severe symptoms the next day are associated with negative values

In both cohorts, multivariate logistic regression indicated that the rest‐activity *I* < *O* parameter was significantly and independently associated with global quality of life (EORTC questionnaire) and interference with enjoyment of life (MDASI questionnaire), alongside all the other selected PROMs (*P* < 0.0001 in both instances). Subgroup analyses according to sex, PS (0 vs 1 vs 2), and age (median‐split) in cohort #1 consistently produced relationships between *I* < *O* on the one hand, and fatigue, anorexia, global quality of life, physical and social functioning on the other hand (Figure 3A).

In cohort #2, day‐to‐day *I* < *O* changes spanned between −17.0% and +9.1% (median: 0) and the intermediate tercile included instances with changes within ±0.3%. The dynamics for a day to the next confirmed an increased severity of fatigue, anorexia, interference with activity, relations, and enjoyment when *I* < *O* worsened during this same time frame (Figure 3B). Altogether, decreased symptom severity was also observed whenever *I* < *O* improved, except for interference with relations with others (Figure 3B). Although statistical significance was not always reached, the trend of the associations in changes appeared consistent.

## 4 DISCUSSION

The results from two subjective PROMs questionnaires (EORTC QLQ‐C30 and MDASI) were compared to circadian rhythm quantitative estimates computed from wrist actigraphy records in two cohorts of cancer patients. Statistically significant and clinically meaningful associations were found between circadian rest‐activity rhythm alterations, and the severity of fatigue and anorexia, as well as the impairment of physical and social dimensions of HR‐QoL and that of general well‐being. Indeed, the size of the differences was substantial and medically meaningful.^40^, ^41^ Moreover, the relationships between circadian rhythms and fatigue or anorexia were strikingly similar in both populations, in which symptoms and HR‐QoL were assessed using distinct questionnaires. This observation, particularly when taking into account the time difference between the two data group collection, as well as complementary subgroup and intrasubject dynamic analyses, further supported the reliability of the findings. However, circadian rhythm alterations were only weakly associated with subjective sleep complaints, in line with prior reports.^43^, ^44^ This underscores the importance of obtaining objective as well as subjective reports of sleep quality and quantity.^45^, ^46^

One of our study's limitations is that it does not provide definitive evidence as to whether circadian rhythm disruption is a cause, a consequence, or a correlate of fatigue and anorexia.^47^ However, fatigue, anorexia, sleep disturbance, and mood alteration, a symptom cluster here linked to altered circadian rhythm, also characterize both jet lag after long‐haul transmeridian flights and shift work, two conditions causing circadian disruption.^8^, ^9^, ^10^, ^11^ This observation therefore supports a causality link between circadian disruption and systemic symptoms, as well as reciprocal interactions.

Circadian rhythms rhythmically regulate physical fitness, appetite, mood, and sleep, while in turn, physical exercise, timing of meals and eating, sleep quality, and duration can reinforce circadian rhythms. Thus, poor circadian entrainment can also be expected in patients suffering from severe fatigue, anorexia, physical deterioration, or social impairment.^48^, ^49^ In contrast, those patients with mild or no fatigue or physical impairment, good appetite and regular social life, likely perform some outdoor activity, eat meals at consistent times, and routinely interact with others, thus better synchronizing their CTS (Figure S3). This hypothesis, supported by the current findings and previous reports,^42^, ^50^, ^51^ has two clinically relevant implications. First, interventions developed to resynchronize subjects experiencing jet lag or shift work ought to be tested in symptomatic cancer patients with circadian disruption, aiming to improve their symptoms through a more robust circadian entrainment.^52^, ^53^ Recent data on behavioral treatments for cancer‐related fatigue or insomnia endorse such novel therapeutic approaches.^54^, ^55^ However, potentially modifiable determinants of circadian disruption need to be identified on an individual basis, as synchronization interventions will require a personalized approach. Second, with the recent rapid development of wearable biosensors, it is possible to implement a continuous remote real‐time monitoring of relevant behavioral and physiological rhythms. Together with the use of electronic PROMs, this could provide more effective care with timely personalized interventions for cancer patients in their home environment.^30^, ^56^, ^57^, ^58^

In conclusion, we found a consistent and robust association between objectively assessed circadian rest‐activity rhythm and fatigue, anorexia, physical and social functioning, as well as global quality of life, primarily in patients with advanced or metastatic gastro‐intestinal cancer. The patients were from different institutions, and PROMs were assessed using two distinct internationally validated questionnaires.^32^, ^33^ The study confirmed and extended the clinical relevance of the dichotomy index *I* < *O*, a circadian parameter that is computed from wrist actigraphy monitoring time series. Here, we showed that *I* < *O* was an objective and continuously assessable biomarker of selected PROMs, which contributed with additional information to HR‐QoL, as well as being an independent prognostic factor of overall survival in cancer patients.^29^, ^31^, ^35^, ^36^, ^37^ The results support the development and testing of interventions targeting the circadian clock to relieve drug‐refractory systemic symptoms and improve HR‐QoL in cancer patients.

## CONFLICT OF INTEREST

Innominato P.F., Komarzynski S., Palesh O.G., Dallmann R., Ulusakarya A., Bouchahda M., Haydar M., Ballesta A., Karaboué A., and Wreglesworth N.I. declare no conflict of interest. Bjarnason G.A. declares Honoraria from Pfizer, Novartis, and Bristol‐Myers Squibb; consulting or advisory role for Pfizer, Novartis, and Bristol‐Myers Squibb; research funding from Pfizer and Merck for his institution; and travel, accommodation expenses from Pfizer and Novartis. Giacchetti S. declares Honoraria from Novartis; consulting or advisory role for EISAI; and travel, accommodation expenses from Roche and Novartis. Spiegel D. declares consulting relationships with Sanofi Aventis and Bristol Meyers Squibb, unrelated to the content of this study. Lévi F.A. declares Honoraria from Philips Respironics; research funding from Philips Respironics for his institution; and travel, accommodation expenses from Philips Respironics and Merck‐Serono.
