# Supplementary figures and images for: Circadian rest‐activity rhythm as an objective biomarker of patient‐reported outcomes in patients with advanced cancer
Source: Cancer Med. 2018 Aug 7;7(9):4396–405. doi: 10.1002/cam4.1711 (PMC6143939; doi:10.1002/cam4.1711)

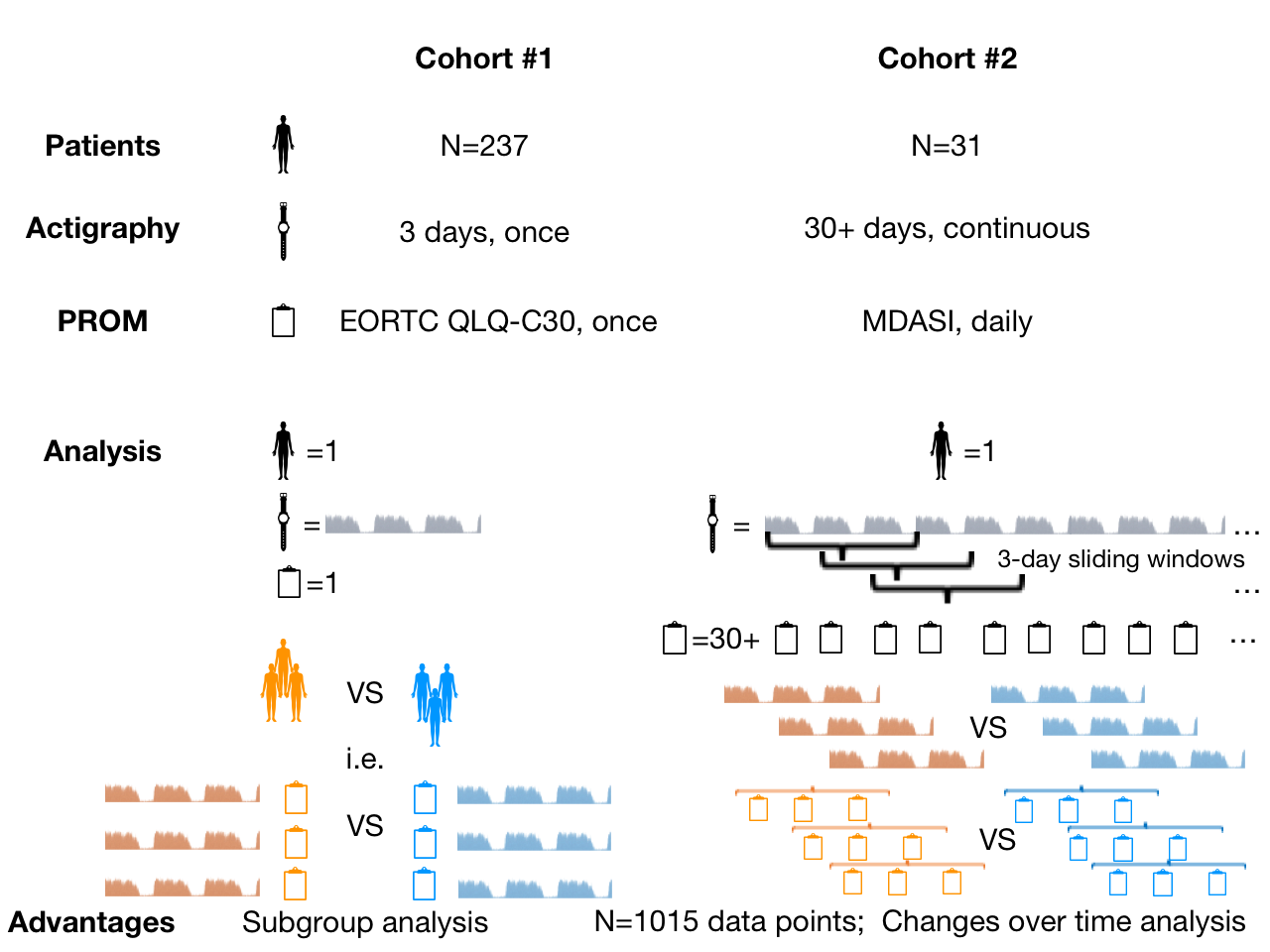

Supplement: Supplementary file 1 [file CAM4-7-4396-s001.tiff]

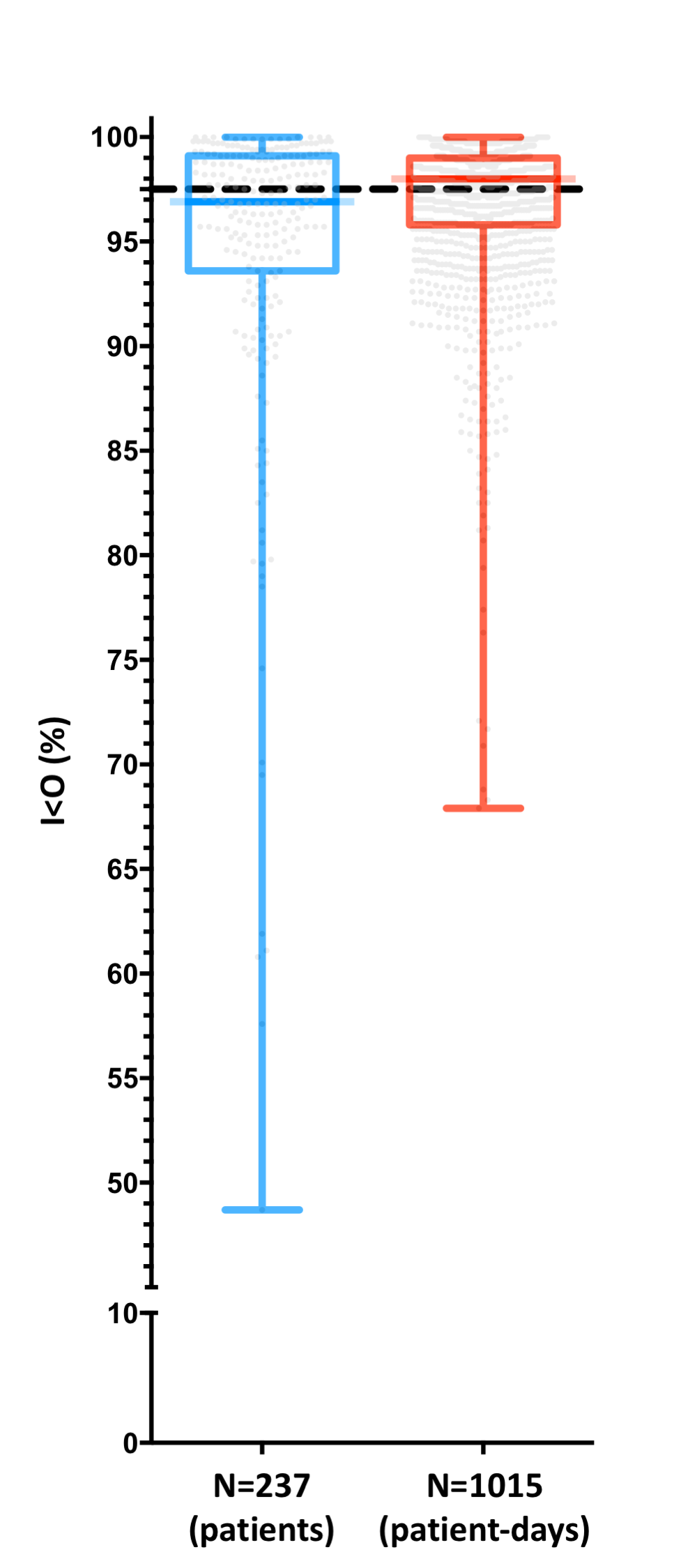

Supplement: Supplementary file 2 [file CAM4-7-4396-s002.tiff]

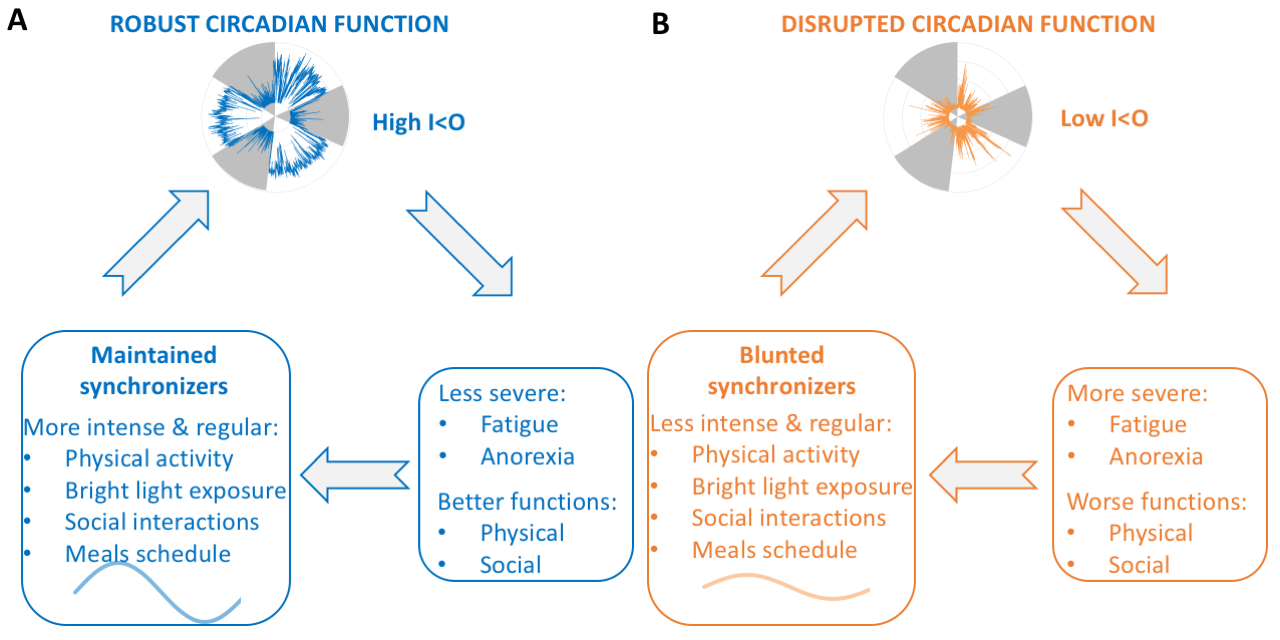

Supplement: Supplementary file 3 [file CAM4-7-4396-s003.tiff]
