# Supplementary material for: Circadian rest‐activity rhythm as an objective biomarker of patient‐reported outcomes in patients with advanced cancer
Source: Cancer Med. 2018 Aug 7;7(9):4396–405. doi: 10.1002/cam4.1711 (PMC6143939; doi:10.1002/cam4.1711)
Supplement: Supplementary file 4 [file CAM4-7-4396-s004.docx]

# **Supplementary Material**

# **Supplementary Tables**

**Supplementary Table 1**

Clinical features of the two study populations: cohort #1 (EORTC questionnaire) and cohort #2 (MDASI questionnaire).

| **Demographic and Medical Variables** | |  | |
| --- | --- | --- | --- |
|  |  | **Cohort #1 (EORTC questionnaire; N=237 patients)** | **Cohort #2 (MDASI questionnaire; N=31 patients)** |
|  |  | **N (%)** | |
| **Age (years)** | **Median** | 60 | 61 |
|  | **Range** | 21-78 | 35-91 |
| **Gender** | **Women** | 89 (37.6) | 14 (45) |
|  | **Men** | 148 (62.4) | 17 (55) |
| **PS (WHO scale)** | **0** | 141 (59.5) | 9 (29) |
|  | **1** | 79 (33.3) | 11 (36) |
|  | **2+** | 17 (7.2) | 4 (12) |
|  | **Unknown** | 0 | 7 (23) |
| **Number of metastatic sites** | **0** | 0 | 7 (23) |
|  | **1** | 107 (45.1) | 24 (77) |
|  | **2** | 89 (37.6) | 0 |
|  | **≥3** | 41 (17.3) | 0 |
| **Site of primary tumor** | **Colon** | 171 (72.2) | 8 (26) |
|  | **Rectum** | 66 (27.8) | 4 (13) |
|  | **Pancreas** | 0 | 9 (29) |
|  | **Breast** | 0 | 5 (16) |
|  | **Other** | 0 | 5 (16) |
| **Prior chemotherapy for metastatic disease** | **No** | 154 (65.0) | 14 (45) |
|  | **Yes** | 83 (35.0) | 17 (55) |
| **Number of patient-days per patient** | **Median (range)** | Not Applicable | 37 (17-155) |

**Supplementary Table 2**

Mean and standard deviation (SD) values of the selected EORTC QLQ-C30 and MDASI items, in cohort #1 and #2, respectively.

| **EORTC items [0-100] (cohort #1)** | | |  | **MDASI items [0-10] (cohort #2)** | | |
| --- | --- | --- | --- | --- | --- | --- |
|  | **Mean** | **SD** |  |  | **Mean** | **SD** |
| **Fatigue** | 37.7 | 25.7 |  | **Fatigue** | 3.96 | 2.27 |
| **Anorexia** | 23.9 | 32.5 |  | **Anorexia** | 2.34 | 2.43 |
| **Sleep trouble** | 34.6 | 30.9 |  | **Sleep disturbance** | 2.18 | 2.08 |
| **Pain** | 22.0 | 25.8 |  | **Pain** | 2.46 | 2.68 |
| **Global Quality of Life** | 60.2 | 21.4 |  | **Interference with Enjoyment of Life** | 3.65 | 2.29 |
| **Physical Functioning** | 76.5 | 25.7 |  | **Interference with Activity** | 4.37 | 2.44 |
| **Social Functioning** | 70.2 | 30.0 |  | **Interference with Relations with Others** | 2.50 | 2.19 |
| **Role Functioning** | 70.6 | 32.4 |  | **Interference with Work** | 4.37 | 2.44 |

**Supplementary Table 3**

Range of PROMs values in the terciles of their distribution. For the EORTC anorexia scale, the data could be split into two groups only.

| **PROMs items** | **Terciles** | | |
| --- | --- | --- | --- |
|  | **1st** | **2nd** | **3rd** |
|  |  |  |  |
| **EORTC [0-100]** |  |  |  |
| Fatigue | 0-22.22 | 27.78-44.44 | 55.56-100 |
| Anorexia | 0 | NA | 33.33-100 |
| Sleep Trouble | 0 | 33.33 | 66.67-100 |
| Pain | 0 | 16.67 | 33.33-100 |
| Global Quality of Life | 0-50 | 58.33-66.67 | 75-100 |
| Physical Functioning | 0-60 | 75-90 | 100 |
| Social Functioning | 0-50 | 66.67-83.33 | 100 |
| Role Functioning | 0-50 | 66.67-83.33 | 100 |
|  |  |  |  |
| **MDASI [0-10]** |  |  |  |
| Fatigue | 0-2 | 3-5 | 6-10 |
| Anorexia | 0 | 1-3 | 4-10 |
| Sleep Disturbance | 0 | 1-2 | 3-9 |
| Pain | 0 | 1-3 | 4-10 |
| Interference with Enjoyment | 0-2 | 3-4 | 5-10 |
| Interference with Activity | 0-3 | 4-5 | 6-10 |
| Interference with Relations | 0 | 1-3 | 4-9 |
| Interference with Work | 0-3 | 4-5 | 6-10 |

NA: not available

**Supplementary Figures**

**Supplementary Figure 1**.

Study flowchart, highlighting the specificities of the datasets gathered in each patient cohort.

**Supplementary Figure 2.**

Distribution of I<O values in the two study populations: cohort #1 (EORTC questionnaire; light blue) and cohort #2 (MDASI questionnaire; red). Boxplots represent median (longer line) and 1^st^ and 3^rd^ quartiles (boxes), while bars show the whole range of values. Raw data points are in light grey. The cut-off value of 97.5% is highlighted with the striped black line.

**Supplementary Figure 3.**

Conceptual framework of the bidirectional relationships between circadian function, systemic symptoms, and external synchronizers. Panel A: in case of higher I<O, patients experience less severe symptoms and better performance functions, positively impacting on the maintenance of regular and strong time cues, hence resulting in more robust synchronization of the body clock. Panel B: in case of lower I<O, more severe symptoms and worse functions occur, inducing behaviors associated with blunted time cues, which ensue an even weaker synchronization of the circadian timing system.
